# Plausibility of Menstrual Cycle Apps Claiming to Support Conception

**DOI:** 10.3389/fpubh.2018.00098

**Published:** 2018-04-03

**Authors:** Alexander Freis, Tanja Freundl-Schütt, Lisa-Maria Wallwiener, Sigfried Baur, Thomas Strowitzki, Günter Freundl, Petra Frank-Herrmann

**Affiliations:** ^1^Department of Gynecological Endocrinology and Fertility Disorders, University of Heidelberg, Heidelberg, Germany; ^2^UniKid, Women’s University Hospital of Düsseldorf, Düsseldorf, Germany; ^3^Department of Obstetrics and Gynecology, University Clinic of Munich, Munich, Germany; ^4^Section Natural Fertility, German Society of Gynecological Endocrinology and Fertility Medicine (DGGEF e.V.), Heidelberg, Germany

**Keywords:** cycle apps, fertility apps, fertility awareness-based methods, natural family planning, fertile window

## Abstract

The interval of peak fertility during the menstrual cycle is of limited duration, and the day of ovulation varies, even in women with fairly regular cycles. Therefore, menstrual cycle apps identifying the “fertile window” for women trying to conceive must be quite precise. A deviation of a few days may lead the couple to focus on less- or non-fertile days for sexual intercourse and thus may be worse than random intercourse. The aim of the present investigation was to develop a scoring system for rating available apps for determining the fertile window and secondarily pilot test 12 apps currently available in both German and English (consisting of 6 calendar-based apps: Clue Menstruations- und Zykluskalender, Flo Menstruationskalender, Maya-Mein Periodentracker, Menstruationskalender Pro, Period Tracker Deluxe, and WomanLog-Pro-Kalender; 2 calculothermal apps: Ovy and Natural Cycles; and 4 symptothermal apps: myNFP, Lady Cycle, Lily, and OvuView). The calendar-based apps were investigated by entering several series of cycles with varying lengths, whereas the symptom-based apps were examined by entering data of cycles with known temperature rise, cervical mucus pattern, and clinical ovulation. The main criteria for evaluating the cycle apps were as follows: (1) What methods/parameters were used to determine the fertile window? (2) What study results exist concerning that underlying method/parameters? (3) What study results exist concerning the app itself? (4) Was there a qualified counseling service? The calendar-based apps predicted the fertile days based on data of previous cycles. They obtained zero points in our scoring system, as they did not comply with any of the evaluated criteria. Calculothermal apps had similar deficits for predicting the most fertile days and produced suboptimal results (Ovy 3/30 points and Natural Cycles 2/30 points). The symptothermal apps determined the fertile days based on parameters of the current cycle: Lady Cycle scored 20/30 points, myNFP 20/30 points, Lily 19/30 points, and OvuView 11/30 points. We concluded that the available cycle apps vary according to their underlying scientific quality and clear rating criteria have been suggested. Three of the tested apps were judged to be eligible for further study. The scientific evaluation of cycle apps depends on good prospective studies undertaken by independent investigators who are free of commercial bias.

## Introduction

The market for mobile applications (“apps”) is growing rapidly and has developed into a huge component of the software industry. Recently, the industry has discovered the female cycle and the prediction of the “fertile window”. Women trying to conceive therefore use those apps to time sexual intercourse.

Most couples trying for pregnancy attempt to detect their fertile window in some fashion, but not all of the available methods provide useful and accurate information. An Australian study that followed 282 patients seeking subfertility care from assisted reproductive technology clinics found that 87% actively tried to improve their fertility awareness knowledge using one or more information resources and that most believed that they had targeted sexual intercourse during the fertile window. However, only 12% of the participating couples were able to correctly identify the fertile window (1, 2). Most of the participants significantly overestimated the duration of the fertile phase. This research suggests that inaccurate timing of sexual intercourse may be a reason for delay in conception or ongoing subfertility, indicating the need for accurate fertility awareness education (3–6).

The requirements for a reliable menstrual cycle app are determined by the physiology of the menstrual cycle. The fertile window is limited by the survival times of the sperm and egg in the genital tract to 4–6 days per cycle. The probability of conception varies within the fertile window, from 5 to 10% on the first 2 days to a maximum of 20–30% during 2 or 3 days before ovulation (7–9). The day of ovulation varies, even in women with fairly regular cycles. In addition, over half of the women present with a variation of more than 7 days within a year (10–12). Therefore, the American Society for Reproductive Medicine recommended recently having sexual intercourse every 1–2 days throughout the whole cycle in order to optimize the natural fertility potential (13). The identification of the fertile window, especially the short window of peak fertility, would represent a helpful tool for couples trying to conceive.

To address this need, a rapidly growing number of apps for mobile phones and tablets claim to help women track their menstrual cycle and identify the fertile window and the window of peak fertility. These apps entice the user to input the most intimate details of their lives, such as their mood, sexual activity, physical activity, physical symptoms, height, weight, and more.

The currently available cycle apps can be classified based on the underlying method used to determine or predict the fertile window into the following groups:

– Apps predicting the fertile days based on previous cycles (former cycle lengths or temperature rise in former cycle): calendar-based apps and calculothermal apps.
– Apps determining the fertile days based on parameters of the current cycle, fertility awareness based (FAB) apps. Apps relying on the symptothermal methods (STMs) use two parameters tracked by the woman herself.

Apps to track the menstrual cycle are required to be quite precise as a deviation of a few days may lead to focusing sexual intercourse on less- or non-fertile days and thus may be worse than random intercourse.

Another major problem is that these apps are not subject to regulation and thus do not provide obligatory and adequate quality control. The user is commonly unable to discern the exact methods and algorithms used by the underlying app.

Tools are needed to assess these apps, primarily for their accuracy for indicating the fertile window. Moglia et al. tried to classify these apps and rated them as being accurate if they predicted the fertile window on the basis of the average length of three preceding cycles (14). However, this approach does not sufficiently account for the physiological variation of the menstrual cycle. Duane et al. focused on the accuracy and evidence for the underlying method (15), but in the case of the known FAB methods, classification should be done according to their efficacy (16–18). There are few highly effective FAB methods, several with medium or lower efficacy, and some with unknown efficacy. Manhart et al. suggested a rating of fertility awareness-based methods (FABMs) that merges the methods with high and medium efficacy into one category (effective methods) (18).

The present study aims to assess the plausibility of cycle apps that claim to support women trying to conceive by considering the state-of-the-art.

We therefore developed a scoring system to rate cycle apps and used it to assess 12 apps that are available in German and English language versions. This evaluation did not reveal the efficacy of the different apps, but selected those that are candidates for inclusion in a prospective trial to determine their efficacy.

## Materials and Methods

We included 12 apps (Table 1).

**Table 1:** Cycle Apps included based on their category.

Category	Apps
Calendar-based apps	Clue Menstruations- und Zykluskalender Flo Menstruationskalender Maya-Mein Periodentracker Menstruationskalender Pro Period Tracker Deluxe WomanLog-Pro-Kalender
Calculothermal apps	Ovy Natural Cycles
Symptothermal apps	Lady Cycle Lily myNFP OvuView

### Inclusion Criteria

The cycle apps had to:

– be available in both German and English language versions
– be most frequently downloaded in one software version
– indicate to be useful for women trying to conceive
– be usable without a connected gadget.

Both types of apps should be included: apps predicting the fertile days on the basis of previous cycle data and apps determining the fertile days based on parameters of the current cycle.

### Description of the Apps

Calendar-based apps predict the fertile days based on the previous cycle lengths. Some of the calendar-based apps use semi-quantitative tests to indicate ovulation [e.g., the determination of urinary luteinizing hormone (LH)]. However, these additional testing features are not included in the free versions and the basic form of the apps can be used without additional tools.

Even the calculothermal apps predict the fertile days based on data of previous cycles: first based on previous cycle lengths and later based on previous temperature shifts. These apps indicate different levels of fertility. In order to investigate their eligibility for couples trying to conceive, we rated their predictive value for the highly fertile days.

Among the apps determining the fertile days based on parameters of the current cycle (FAB-apps) we found only symptothermal apps on the German market, with the exception of OvuView. Lady Cycle and myNFP are based on the Sensiplan method. Lily and OvuView offer different algorithms that can be chosen by the users. Women using the Lily app can choose from three different algorithms that are available. We rated the Lily app using the evidence-based Sensiplan method. OvuView offers 17 different methods to their users. We evaluated a symptothermal combination (Billings/Rötzer).

### Evaluation of the Apps

We evaluated the apps with regard to their plausibility to support women trying to conceive in two ways:

1. *Scoring system* according to different criteria
2. *Pilot testing* by entering menstrual cycle data into each of the selected apps to see how the app acts.

#### Scoring System

We rated each app according to eight criteria, which were weighted based on their relevance for achieving pregnancy (Table 2). The rating would be somewhat different for contraceptive use.

**Table 2:** Scoring system.

Evaluation criteria		Points
Considering an estrogen parameter and/or LH to determine the fertile window	Cervical mucus	5
	Other estrogen parameters	1–3
	Urinary estrogen level	2
	Urinary luteinizing hormone	2
	No	0

Considering a progesterone parameter to determine the fertile window	Basal body temperature (BBT, Holt rule, “3 over 6”)	3
	BBT (classical coverline or untested algorithm)	1
	Other progesterone parameters	1–3
	No	0

Which algorithm is used to determine the fertile window	Symptothermal method	3
	Cervical mucus method	2
	Classical calendar method	1
1-8	Calculations (insufficient)	0
	Unknown	0

Efficacy of the underlying FAB method (referring to the S1-guideline of the German Society for Gynecological Endocrinology and Fertility Medicine, DGGEF)	Category 1	3
	Category 2	3
	Category 3	0
	Category 4	0

Study quality regarding the underlying methods/algorithms	Good	4
	Mediocre	1
	Not available or insufficient	0

Study quality regarding the app	Good	4
	Mediocre	1
	Not available or insufficient	0

Origin of data belonging to the app	Independent institutions	4
	Enterprise itself	1
	No data	0

Is there any qualified FAB-counseling?	Yes	3
	Fair instructions in the app	1

*The maximum of points that could be obtained were 30 points*.

The main criteria of our scoring system are as follows:

– What methods/parameters are used to determine the fertile window?
– What study results exist concerning that underlying method/parameters?
– What studies have been done of the app itself?
– Is there a qualified counseling service?

The rationale of the criteria in detail:

*Criterion 1*: To achieve a high score in this category, an algorithm/app has to be based on an evidence-based estrogen parameter, because this is the most accurate way to prospectively identify the highly fertile days. Among the estrogen parameters, the cervical mucus symptom gets the highest rating because in addition to being an estrogen parameter, the presence and quality of the cervical mucus is an essential determinant of sperm survival. Cervical mucus is observed externally at the vulva and its characteristics are evaluated daily by the user. Other estrogen parameters (e.g., autopalpation of the cervix and semiquantitative estrogen levels in the urine) get fewer points than the cervical mucus symptom because they are more variable or are less mature or have no direct correlation to sperm survival (19, 20). The LH parameter is rated less strongly as it gets positive at ovulation and thus may not cover all of the highly fertile days that precede it.
*Criterion 2*: The progesterone parameter is useful for women trying to conceive because it confirms that ovulation has occurred and indicates when to expect menstrual bleeding or a positive pregnancy test. This observation is notably important for women with irregular cycles who are trying to conceive. In comparative studies (using different algorithms), it was found that the Holt rules, which are used by different FABM groups, reduced the percentage of non-analyzable cycles compared to classical coverlines (20, 21). The progesterone marker is not useful for predicting the fertile phase.
*Criterion 3*: An optimal method should cover the complete fertile window and allow detection of the rising fertility within the fertile window. We rated the different STMs equally, as a double-check to determine the beginning of the fertile phase is not necessary for achieving pregnancy (cervical mucus alone is sufficient as well) in contrast to the situation of wanting to avoid pregnancy. Classical calendar methods use 10–12 previous cycles to predict the fertile window based on the whole range of observed cycle lengths. Therefore, the fertile window usually is quite large but covers the fertile phase to a great extent. “Insufficient” calculations use average cycle length or the length of a single preceding cycle.
*Criterion 4*: Most of the studies were performed to evaluate FABMs contraceptive efficacy and not the supporting effect for women trying to conceive. If a method has proven to be effective for avoiding pregnancy, it implies that it covers the complete fertile window. FAB methods can be classified according to their method efficacy (unintended pregnancy rates per 100 women years): very effective < 1, medium effective 1–4, less effective > 4, and unknown.
*Criterion 5*: There are underlying methods both with and without previous scientific studies. An adequate effectiveness study is prospective, non-parametric, measures real pregnancy rates, uses a clear pregnancy classification, valid statistical methods, includes a sufficient number of women, and has a low percentage of participants lost to follow-up. In addition, correlation studies (e.g., correlation of self-observed parameters with ovulation determined by ultrasound) and time-to-pregnancy studies are considered for this criterion as well.
*Criterion 6*: It is important that the app has been evaluated adequately. The study quality concerning an app should be the same as required for other FABMs. Apps that are advertised to identify the fertile window require studies in both directions as they are used for both purposes, to either achieve or avoid pregnancy.
*Criterion 7*: It is of course necessary that the app is scientifically evaluated by independent investigators who do not have financial interests and will not profit, from the sale of the app.
*Criterion 8*: As with other medical devices, sufficient support for the user has to be available to address problems, e.g., with observing the cycle parameters and to clarify individual situations. Sufficient support means at best that there are trained and certified teachers for the app.

#### Pilot Testing

The pilot testing serves to explore the implementation of the underlying method (in case of known FABMs) or to understand how the algorithm principally works.

Calendar-based apps are investigated using four series of cycles with varying cycle lengths (scenario 1: 9 × 28 and 3 × 32 days; scenario 2: 26, 32, 33, 28, 30, and 34 days; scenario 3: 4 × 30, and 2 × 26 days; and scenario 4: 3 × 28, 56, and 2 × 28 days) (Table 3). The physiological fact that ovulation occurs 14 ± 2 days before the end of the cycle is used to verify the plausibility of the prediction. The calculothermal apps and the symptothermal apps are investigated using three cycles with known temperature rise, cervical secretions, and clinical ovulation (Table 4). The temperature rise is determined according to the Holt rules (10, 21).

**Table 3:** Test scenarios: Predicted day of ovulation.

Scenario 1							
*APP/cycle length*	9*28	**32**	**32**	**32**			
Menstruationskalender Pro		15	18	19	19		
Flo Menstruationskalender		14	14	14	15		
Clue Menstruations- und Zykluskalender		15	15	15	16		
Period Tracker Deluxe		15	16	18	19		
Maya-Mein Periodentracker		14	14	14	14		
WomanLog-Pro-Kalender		15	15	16	16		

**Scenario 2**							
*App/cycle length*	**26**	**32**	**33**	**28**	**30**	**34**	
Menstruationskalender Pro	15	13	16	17	17	17	18
Flo Menstruationskalender	14	12	15	16	16	16	17
Clue Menstruations- und Zykluskalender	15	16	17	17	17	17	17
Period Tracker Deluxe	15	13	16	17	18	17	18
Maya-Mein Periodentracker	14	12	15	16	16	16	16
WomanLog-Pro-Kalender	15	13	16	17	17	17	18

**Scenario 3**							
*APP/cycle length*	4*30	**26**	**26**				
Menstruationskalender Pro		17	16	15			
Flo Menstruationskalender		16	15	15			
Clue Menstruations- und Zykluskalender		17	17	17			
Period Tracker Deluxe		17	16	14			
Maya-Mein Periodentracker		16	15	15			
WomanLog-Pro-Kalender		17	16	16			

**Scenario 4**							
*APP/cycle length*	3*28	**56**	**28**	**28**			
Menstruationskalender Pro		15	15	15	15		
Flo Menstruationskalender		14	14	14	14		
Clue Menstruations- und Zykluskalender		15	15	15	15		
Period Tracker Deluxe		15	24	24	24		
Maya-Mein Periodentracker		14	14	14	14		
WomanLog-Pro-Kalender		15	15	15	15		

*Cycle length is indicated in bold. The predicted day of ovulation for the upcoming cycle is shown in the next column. For example, in scenario 1 after observing 9 consecutive 28 day cycles the apps predicted ovulation in cycle 10 to be on day 14 or 15 depending on the app. After entering cycle 10 (32 days long), the predicted day of ovulation ranged from day 14 to 18 depending on the app*.

**Table 4:** Predicted most fertile days in relation to cycle length/temperature rise.

	Reference cycle 1		Most fertile days	
	Temperature rise	Cycle length	Beginning	End
Reference	14	25	10	14
Natural Cycles^a^			14	17
Ovy^a^			12	17

	**Reference cycle 2**		**Most fertile days**	
	**Temperature rise**	**Cycle length**	**Beginning**	**End**

Reference	19	32	16	19
Natural Cycles^a^			13	16
Ovy^a^			13	18

	**Reference cycle 3**		**Most fertile days**	
	**Temperature rise**	**Cycle length**	**Beginning**	**End**

Reference	22	31	17	22
Natural Cycles^a^			14	17
Ovy^a^			9	14

*^a^A previous cycle length of 30 days was stated by demand of the app. Before entering the first cycle*.

## Results

### Apps Predicting the Fertile Days Based on Data of Previous Cycles

#### Scoring

The six calendar-based apps were rated at a level of zero points in our scoring system, because they did not comply with any of the eight criteria (Table 2). The calculothermal apps had similar deficits for predicting the most fertile days of the cycle and provided suboptimal results (Ovy 3/30 points and Natural Cycles 2/30 points) (Tables 4,5).

Algorithms used by the apps are developed by the corporate entities that own them, and almost all lack any studies documenting their efficacy. The corporate enterprise behind Natural Cycles published data from its users. However, critical comments have been published recently (17, 22). None of the apps provided a link to a qualified FAB counseling service.

#### Pilot Testing

Calendar-based apps and calculothermal apps predict the day of ovulation and the most fertile days by estimating an average cycle length on the basis of as little as one to three previous menstrual cycles, or, if unknown, the length of the prospective cycle may be estimated by asking the woman about general perception of her typical cycle length. But if all else fails, and lacking better data, it is done by assuming a 28-day cycle. Some apps track further parameters such as cervical secretions or basal body temperature (BBT). However, these parameters do not influence the prediction. The calculothermal apps correct the fertile window retrospectively after the temperature rise, when the fertile window has closed.

Calendar-based apps and calculothermal apps seem to predict the fertile window in a three-step-process:

Step 1— Prediction of the onset of the next menstrual period based on the mean cycle length (even if only 1, 2, or 3 previous cycles are available). If no data are available, cycle length was determined by the app itself (28 days) or by the user.
Step 2— Ovulation mostly was fixed 14 days before the predicted upcoming menstrual bleeding (based on the presumption that the luteal phase requires 14 days).
Step 3— The app predicts the fertile window based on the ovulation date, e.g., 4 days before ovulation + day of ovulation + 3 days in the postovulatory phase = 8 days. The exact definition of the fertile window varies between the different apps.

Our scenarios demonstrate that the calendar-based apps misstated the day of ovulation if the cycle length differed from the estimated cycle length in the current cycle (Table 3). Some apps try to alter the predicted date. However, these changes could be insufficient and resulted in overcorrection (Period Tracker Deluxe). Other apps did not change anything or not enough in the current cycle (Maya, Clue).

The calculothermal apps were tested by supplying temperature data from three scenarios (cycles 1–3) (Table 4). Prediction was insufficient as well, as the determination of highly fertile days was performed using data from previous cycles or anamnestic data. The Ovy and Natural Cycles apps did not consider any information from the current cycle either. Therefore, they failed to predict the highly fertile days correctly, when cycle length and ovulation vary by some number of days. None of the apps tested provided sufficient annotations explaining how potentially disturbing factors of temperature should be handled.

### Apps Determining the Fertile Days Based on Parameters of the Current Cycle

#### Results of Scoring

Lady Cycle was scored at 20/30 points, myNFP 20/30 points, Lily 19/30 points, OvuView 11/30 points (Table 5).

**Table 5:** Scoring results of the symptothermal and calculothermal apps.

	Lady cycle	Lily	myNFP	OvuView	Natural cycles	Ovy
Recording of estrogen parameters in the follicular phase	5	5	5	5	0	0
Recording of progesterone parameters in the luteal phase	3	3	3	1	1	3
Which algorithm is used to determine the fertile window?	3	3	3	2	0	0
Efficacy of the underlying fertility awareness-based method	4	4	4	2	0	0
Study quality regarding the underlying methods/algorithms	4	4	4	1	0	0
Study quality regarding the app	0	0	0	0	0	0
Origin of data belonging to the app	0	0	0	0	1	0
Is there any qualified FAB-counseling?	1	0	1	0	0	0

Total	20	19	20	11	2	3

The symptothermal apps determined the fertile window using parameters of the current cycle: including cervical secretions (“estrogen parameter”) and BBT (“progesterone parameter”). Thus, the fertile window was not predicted, but determined during the course of the cycle coinciding with the underlying STM.

None of the apps reached the maximum score. They have yet not been evaluated in a prospective pregnancy study, and so there are no sufficient data available to reach final conclusions. The underlying methods could be rated according to the available studies.

#### Pilot Testing

The Lady Cycle and myNFP apps basically perform their analyses correctly according to the reference method (peak cervical mucus and Holt BBT rules) (Table 5). The Lily app has apparent minor limitations (i.e., concerning descriptions of cervical mucus quality) but indicates the fertile window accurately. The OvuView app indicated the highly fertile days correctly based on the cervical secretion data from the current cycle, if the preselected settings had been entered appropriately.

The overall results of our scoring system are presented in Table 5.

## Discussion

App-guided indication of daily fertility may influence sexual behavior of couples trying to conceive as well those trying to avoid pregnancy. Because of the increasing popularity and usage of these apps, we suggested a scoring system to evaluate how well these apps can indicate the highly fertile days in the woman’s cycle and thus help couples achieve pregnancy (Table 2). The criteria for this scoring system reflect various factors, including the underlying methods or algorithm of the app, whether information from the current cycle is used, the evidence of the underlying algorithm, the availability of adequate scientific studies on the app itself, and whether there is a counseling service for queries of the users.

The main difference among the various menstrual cycle apps is that one group predicts the most fertile days based on data of previous cycles (calendar-based apps and calculothermal apps), whereas the other group determines the fertile days based on parameters of the current cycle (FAB-apps, natural family planning-apps). Specifically, we entered cycle data into the apps to receive an impression how the respective app works.

### Physiological Variability of the Cycle: The Crucial Problem for Predicting the Fertile Days

The tested calendar-based apps and both of the calculothermal apps predict the most fertile days of the cycle, and the day of ovulation, based on a few, averaged data of preceding cycles. However, this retrospective method is viewed as inadequately reliable as a result of the known variability of the cycle length and ovulation day from month to month, a fact that has been known to science since the 1930s (10–12, 23). Our results confirm that the utility of this technique is limited and that the most fertile days are often missed as a result of the normal variation of the cycle length and the changing day of ovulation (Tables 3 and 4).

Before the availability of the oral contraceptive pill, calendar methods were used widely for contraception. Research on the menstrual cycle has been done for 100 years now. In the 1920s, the first calendar methods were developed. The state of the art of reproductive gynecology, even at that time, already demanded that the whole range of the cycle had to be included in order to predict the days that pregnancy could result if coitus occurred in that timeframe during the following cycle. Therefore, the shortest and the longest cycle out of at least 6 (better 12) preceding cycles had to be considered. Of course, this leads to long potentially fertile phases. Nevertheless, the calendar methods have not been proven to be adequately effective for couples desiring reliable contraception (10). Surprisingly, none of the apps consider the full variation of cycle characteristics. With the development and availability of the cycle apps, we now have methods to predict the fertile days for the first time, developed on the basis of accidental data, and disregarding knowledge of the spontaneous individual cycle. Therefore, we conclude that the calendar-based apps and the similarly working calculothermal apps are even less suitable than traditional calendar methods for predicting the most fertile days during the menstrual cycle.

There is the risk that apps predicting the most fertile days based on information of previous cycles may have adverse effects, because if the highly fertile days are incorrectly estimated, it may lead some couples trying to conceive to have sexual intercourse at the wrong time, or influencing other couple’s contraceptive behavior.

### Determining the Fertile Window Using Parameters of the Current Cycle

The evaluated symptothermal apps determine the most fertile days according to parameters observed in the current cycle. The Lady Cycle and myNFP apps, as well as one of the methods offered by the Lily app, are apps based on the Sensiplan method. According to our pilot testing, they correctly determine the fertile window consistently. The OvuView app indicated the woman’s most fertile days correctly based on her cervical secretions. However, in our opinion, a woman has to be an FAB expert in order to be able to preselect the suitable settings properly. Furthermore, the symptothermal rules used do not reflect the original method by Rötzer as indicated by OvuView. It should be noted that this issue is not relevant for achieving pregnancy and would only be important for couples seeking to avoid pregnancy.

For women trying to conceive, the observation of cervical mucus provides the cardinal sign of fertility and has proven to be the most important marker of the most fertile days. Cervical mucus has estrogen-dependent features and predicts ovulation during the course of the menstrual cycle. Its physiological alterations during the menstrual cycle indicate the increasing probability of conception and peak fertility (23–25). Moreover, sperm survival in the vagina and cervix is only possible in the favorable surroundings provided by cervical mucus.

A considerable amount of data suggests that observation of cervical mucus changes can allow close estimates of the day of ovulation. This type of mucus analysis is easy to learn and suitable for a large cross-section of women. Studies correlating the woman’s observations of the cervical mucus to the day of ovulation (determined by daily ultrasonic follicle measurement or daily urinary LH or pregnanediol measurements) as well as studies on the probability of conception on different days of the fertile window have shown that the cervical mucus pattern indicates the few days of peak fertility in the cycle fairly precisely (23–32). The vast majority of women are able to detect it: the World Health Organization findings and other publications show that about 95% of women are able to learn to accurately observe the cervical mucus pattern (10, 33).

Scarpa et al. and Bigelow et al. found that the presence of cervical secretions on the day of intercourse is a better predictor of the likelihood of conception than the timing of intercourse (23, 24). Dunson et al. concluded that the presence of cervical secretion is an even better fertility marker than LH kits, as it identified a larger fertile window and facilitates more frequent intercourse (34). In a probability-of-conception analysis, a European database of cycles showed that those couples with a single episode of sexual intercourse during the fertile time needed a larger number of cycles in order to achieve conception (24). These findings indicate that the observation and monitoring of cervical secretions is a useful way to identify days when there is a high probability of conception if intercourse takes place.

The temperature rise is a progesterone marker and thus confirms ovulation and the ensuing end of the fertile window of the current cycle. However, it cannot be used to fully predict the fertile window. There are different algorithms to interpret the temperature rise (e.g., Holt and Döring). The detection rate of the temperature rise and the correlation with ovulation descends from the underlying algorithm (10, 35). Therefore, new algorithms cannot rely on studies using other algorithms.

### Effectiveness Studies

The Sensiplan method used by the Lady Cycle, myNFP, and one variation of the Lily app is evidence-based and has been the subject of extensive research. A prospective time-to-pregnancy study followed 340 healthy women trying for pregnancy using that method from their first cycle. The pregnancy rates after 1, 3, 6, and 12 cycles were 38, 68, 81, and 92% respectively (36). For contraceptive use, the pregnancy rate was 0.4/100 women/year when no intercourse occurred in the fertile time. It was therefore comparable to other effective family planning methods, provided the guidelines were consistently followed (37, 38). The data from these studies cannot be extrapolated to other STMs.

There are no efficacy studies on any of the symptothermal apps themselves so far. Therefore, in the present evaluation none of the apps was rated with the highest score in this category.

Although the developers of the Natural Cycles app published results from their model (39–41), the study designs and data have severe limitations (including insufficient details such as detection of unplanned pregnancies and use of highly inappropriate methods to calculate efficacy), and a critique in a letter-to-the-editor (22) contends that estimates of the rates of unplanned pregnancy that were provided cannot be considered reliable. In contrast, the developers argue that the app has been certified. However, the certification they are relying on, from a German association for technical inspection (TÜV Süd), does not say anything about the efficacy of that app for family planning.

### Learning to Observe Cycle Parameters—The Input of the Woman Is Still Necessary

Today’s methods that have proven efficacy still require the observation of certain cycle parameters by the individual woman. This necessitates effective in-app guidance and a qualified counseling service for queries from the users. The learning process with STMs mainly entails making valid observations of the cervical secretions and identifying disturbed temperature values. This should also be subject of future studies on app effectiveness.

### New Developments

In 1965, Hartmann wrote: “Wanted: an easily detected sign of impending ovulation” (10). Since the 1950s, many different biochemical and physical parameters have been tested to investigate their suitability for determining the fertile days of the menstrual cycle. Some apps developed for this aim use new parameters (such as end-expiratory carbon dioxide pressure, pulse rate, and others). Frequently, some correlation of these new parameters with cycle phases can be detected. However, at this time, the correlations have not been precise enough to allow us to develop new and reliable methods of fertility tracking and to replace present natural family planning methods. In any case, finding a new, easily detected parameter remains a desirable goal.

### Limitations of This Evaluation

With our plausibility rating, we can identify cycle apps that hold promise for further studies. It also allows us to exclude other apps that are not plausible according to the medical state of the art. However, we cannot make any statements on the effectiveness of the plausible apps. Furthermore, we did not evaluate privacy, data protection, security, and costs of the apps. Our evaluation focuses on the plausibility of cycle apps that are marketed to be used to achieve pregnancy. Our rating cannot be transferred to the question of contraceptive efficacy. If women use the apps for contraception, the determination of the outside limits of the fertile window is required to decrease the chances of unintended pregnancy (6, 16). Our evaluation includes apps available in German and English and therefore represents only a small percentage of the fertility apps available in English.

## Conclusion

As a result of the physiological variations of the menstrual cycle length and ovulation day, the fertile window cannot be predicted easily, and it has to be determined during the ongoing cycle. Therefore, to help couples wishing to conceive, apps based on data from previous cycles alone are not suitable to indicate the most fertile days. A preselection of cycle apps along the state-of-the-art and according to clear criteria has been suggested. Apps eligible for further study were identified. The scientific evaluation of these cycle apps will depend on good prospective studies undertaken by researchers independent of the commercial interests of the companies. Women’s health is big business. That is why we expect to see more and more menstrual cycle apps flooding the market in the future.

## Author Contributions

AF and PF-H: protocol development, data evaluation, data analysis, and manuscript writing. TF-S: score development, data analysis, and manuscript writing. L-MW: data evaluation, data analysis, and manuscript drafting. SB: protocol development, data analysis, and manuscript editing. GF: protocol development, score development, data analysis, and manuscript editing. TS: protocol development and data analysis.

## Conflict of Interest Statement

The authors declare that the research was conducted in the absence of any commercial or financial relationships that could be construed as a potential conflict of interest.
